# On the adsorption and reactivity of element 114, flerovium

**DOI:** 10.3389/fchem.2022.976635

**Published:** 2022-08-25

**Authors:** A. Yakushev, L. Lens, Ch. E. Düllmann, J. Khuyagbaatar, E. Jäger, J. Krier, J. Runke, H. M. Albers, M. Asai, M. Block, J. Despotopulos, A. Di Nitto, K. Eberhardt, U. Forsberg, P. Golubev, M. Götz, S. Götz, H. Haba, L. Harkness-Brennan, R.-D. Herzberg, F. P. Heßberger, D. Hinde, A. Hübner, D. Judson, B. Kindler, Y. Komori, J. Konki, J.V. Kratz, N. Kurz, M. Laatiaoui, S. Lahiri, B. Lommel, M. Maiti, A. K. Mistry, Ch. Mokry, K. J. Moody, Y. Nagame, J. P. Omtvedt, P. Papadakis, V. Pershina, D. Rudolph, L.G. Samiento, T.K. Sato, M. Schädel, P. Scharrer, B. Schausten, D. A. Shaughnessy, J. Steiner, P. Thörle-Pospiech, A. Toyoshima, N. Trautmann, K. Tsukada, J. Uusitalo, K.-O. Voss, A. Ward, M. Wegrzecki, N. Wiehl, E. Williams, V. Yakusheva

**Affiliations:** ^1^ GSI Helmholtzzentrum für Schwerionenforschung, Darmstadt, Germany; ^2^ Helmholtz-Institut Mainz, Mainz, Germany; ^3^ Johannes Gutenberg-Universität Mainz, Mainz, Germany; ^4^ Japan Atomic Energy Agency, Tokai, Japan; ^5^ Lawrence Livermore National Laboratory, Livermore, CA, United States; ^6^ Lund University, Lund, Sweden; ^7^ RIKEN, Wako, Japan; ^8^ University of Liverpool, Liverpool, United Kingdom; ^9^ Australian National University, Canberra, ACT, Australia; ^10^ University of Jyväskylä, Jyväskylä, Finland; ^11^ Saha Institute of Nuclear Physics, Kolkata, India; ^12^ Indian Institute of Technology Roorkee, Roorkee, India; ^13^ University of Oslo, Oslo, Norway; ^14^ Łukasiewicz Research Network—Institute of Electron Technology, Warsaw, Poland

**Keywords:** nuclear chemistry, radiochemistry, superheavy elements, element 114, adsorption, recoil separators

## Abstract

Flerovium (Fl, element 114) is the heaviest element chemically studied so far. To date, its interaction with gold was investigated in two gas-solid chromatography experiments, which reported two different types of interaction, however, each based on the level of a few registered atoms only. Whereas noble-gas-like properties were suggested from the first experiment, the second one pointed at a volatile-metal-like character. Here, we present further experimental data on adsorption studies of Fl on silicon oxide and gold surfaces, accounting for the inhomogeneous nature of the surface, as it was used in the experiment and analyzed as part of the reported studies. We confirm that Fl is highly volatile and the least reactive member of group 14. Our experimental observations suggest that Fl exhibits lower reactivity towards Au than the volatile metal Hg, but higher reactivity than the noble gas Rn.

## 1 Introduction

The superheavy elements (SHE, Z ≥ 104) are accessible only at the level of single atoms. Their production rates decrease rapidly and their nuclear lifetimes drop with increasing atomic number, *Z* (Oganessian and Utyonkov, 2015). This makes chemical studies of SHE very challenging (Türler et al., 2015; Türler, 2016). Relativistic effects strongly affect the electronic shell structure in SHE, and, hence, chemical properties of SHE, rendering their study very interesting. Many theoretical investigations on atomic and chemical properties of the heaviest elements have been performed during the last two decades (Pershina, 2015; Eliav et al., 2016). Among them, flerovium (Fl, *Z* = 114), the heaviest member of group 14, was the focus of intense efforts. Early atomic calculations on superheavy elements suggested Fl in the elemental state to be noble–gas like or a very volatile liquid bound by dispersion (London) forces only (Pitzer, 1975). The spin-orbit splitting of the *7p* shell leads to two sub-shells, *7p*_*1/2*_ and *7p*_*3/2*_. The *7p*_*1/2*_ one is spherical, like the *7s* orbital, and both are strongly relativistically stabilized. In Fl, the large energy gap between the highest fully occupied *7p*_*1/2*_ and the lowest unoccupied *7p*_*3/2*_ sub-orbitals causes the outermost electrons to be almost inaccessible for chemical bonding. An excitation energy of more than 3 eV would be needed for the electron transfer from the ground state ^1^ *S*_0_ to the lowest excited state *P*_1_ (Eliav et al., 2016). Spin-orbit effects strongly weaken the strengths of Fl bonds in compounds. Theoretical studies predict that the Fl tetravalent oxidation state is not favoured and Fl compounds are thermodynamically unstable, except for FlF_2_ and FlO (Liu et al., 2001; Schwerdtfeger and Seth, 2002). Periodic-code calculations based on density functional theory (DFT) suggest Fl to bind loosely upon adsorption on a Au (111) surface (Pershina, 2018). Calculations on bulk properties, including the cohesive energy and the analysis of its relation to the adsorption energy on hetero-surfaces, were performed recently for SHE from Cn to Og (Trombach et al., 2019). Reporting the adsorption energy of Fl on Au (111) to be in the range $E_{ads}^{Au}\left(Fl\right)=\;-\left(0.4-0.9\right)$ eV, as predicted by different approaches, the authors concluded that Fl is in general more reactive than Cn (*Z* = 112) and exhibits an interaction with Au about 0.1 eV stronger than Cn (Trombach et al., 2019). However, the predicted values for the adsorption energy of Fl and Cn on Au (111) are similarly low, and the adsorption is mainly of dispersive nature in both cases.

Flerovium is the heaviest chemical element, for which chemical properties have been investigated experimentally. Fl isotopes can be formed in the nuclear fusion of _20_Ca and _94_Pu. The well-established method of adsorption chromatography was applied to study the interaction strength of single Fl atoms with solid surfaces (Türler et al., 2015). Based on the observation of the nuclear decay chains assigned to three Fl atoms and their progenies, an adsorption enthalpy value of Fl on Au, $-\Delta H_{ads}^{Au}\left(Fl\right)=34_{-11}^{+54}$ kJ·mol^−1^ (95% confidence interval, c.i.), was derived from a first study (Eichler et al., 2010). In further studies, performed by the same collaboration, no further Fl atoms were registered, testifying to the challenging nature of chemical studies of SHE (Türler et al., 2015; Yakushev and Eichler, 2016). A particular problem was the presence of a substantial background in the nuclear spectra due to the presence of unwanted nuclear reaction products inside the detectors, which complicates the unambiguous identification of single Fl atoms. Sources of this background can be efficiently suppressed by a kinematic recoil separator (Düllmann et al., 2005; Wittwer et al., 2010). This technique is applied in all Fl chemistry experiments at the GSI Helmholtzzentrum für Schwerionenforschung GmbH (GSI), Darmstadt, Germany. Here, the gas-filled TransActinide Separator and Chemistry Apparatus (TASCA) serves as a pre-separator (Semchenkov et al., 2008). Two Fl atoms were registered on Au kept at room temperature in a first run, suggesting a metallic Fl−Au interaction (Yakushev et al., 2014). A lower limit of $-\Delta H_{ads}^{Au}\left(Fl\right)>48$ kJ/mol (95% c.i.) was evaluated. Whereas the adsorption enthalpy values from both experiments agree within rather large error bars (Eichler et al., 2010; Yakushev et al., 2014), the different interpretations on the chemical reactivity of Fl drawn from the two studies called for further investigations of the chemical nature of Fl.

Here, we report on the observation of six additional Fl atoms in two further gas chromatography studies on Fl at TASCA and present the analysis and discussion on the chemical reactivity of Fl and its adsorption behaviour on an inhomogeneous Au surface.

## 2 Experimental

The most neutron-rich Fl isotopes, ^288^Fl and ^289^Fl have half-lives of *T*_*1/2*_ = $0.65_{-0.08}^{+0.12}$ s and *T*_*1/2*_ = $2.3_{-0.5}^{+0.8}$ s, respectively (Såmark-Roth et al., 2021), and thus, they are suitable for chemical studies. As in the first TASCA experiment (Yakushev et al., 2014), these isotopes were produced in two further runs at TASCA in the nuclear fusion of ^48^Ca with ^244^Pu followed by the evaporation of three or four neutrons, at compound nucleus excitation energies of 40–44 MeV (Düllmann et al., 2010; Gates et al., 2011; Såmark-Roth et al., 2021).

**FIGURE 1:**
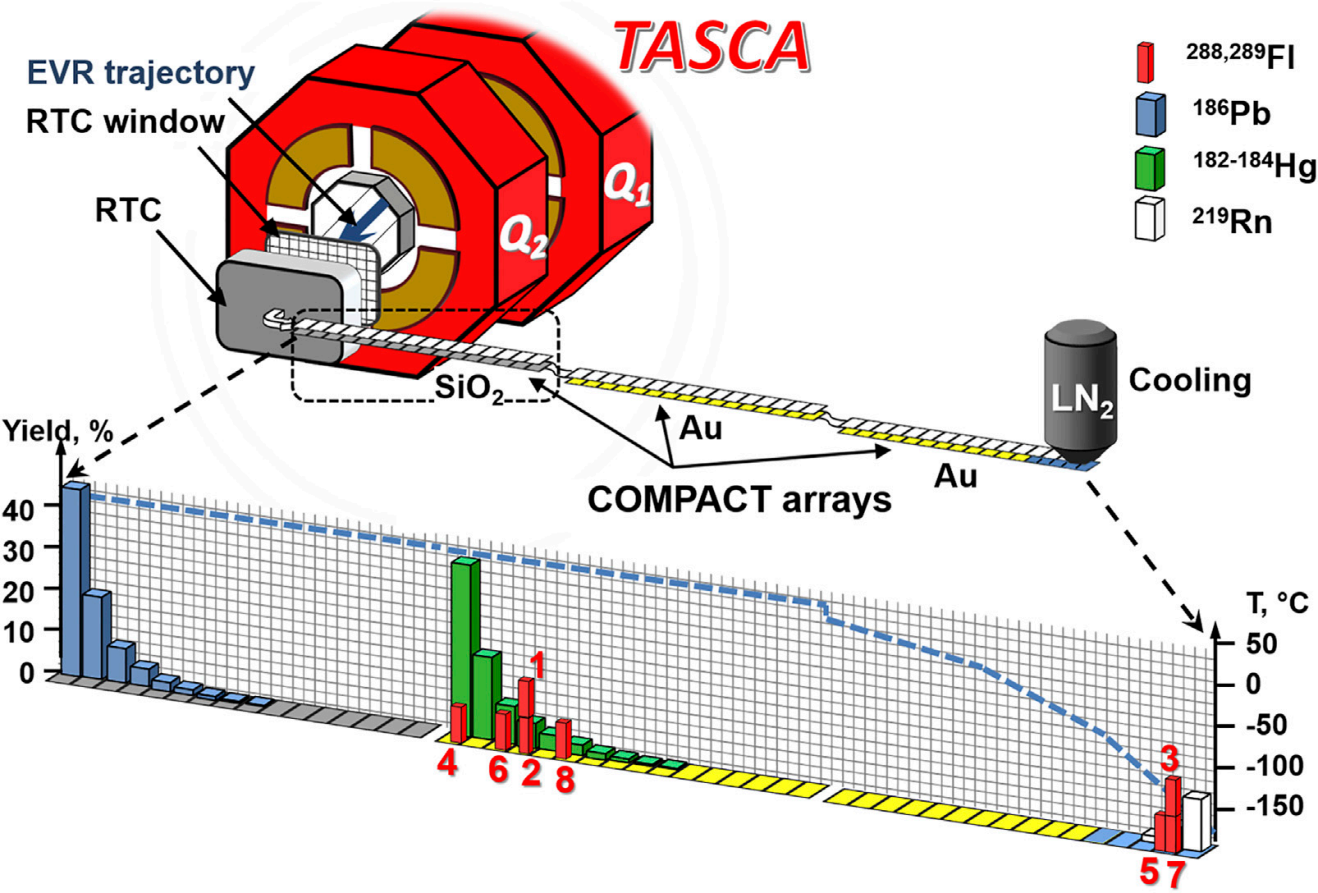
Experimental setup at TASCA and deposition pattern of ^288,289^Fl as well as ^186^Pb, ^182-184^Hg, and ^219^Rn: The detection setup allowed the separation of non-volatile metals (Pb, blue bars) from volatile metals (Hg, green bars) and noble gases (Rn, white bars). The positions of Fl decays observed in three TASCA runs (Yakushev et al., 2014, this work) are shown as red bars. Numbers in red refer to the decay chains presented in Figure 2. The setup consisted of two Au-coated COMPACT arrays (yellow) in the first (Yakushev et al., 2014) and in the last Fl experiment. In the second run, a third COMPACT detector array (indicated by the dashed black rectangle), coated with SiO_2_ (grey), was added in front of the two Au-coated (yellow) arrays. A 5-cm PTFE tube (inner diameter 4 mm) connected the first COMPACT detector with the RTC. The following COMPACT detector(s) was (were) connected via a 30-cm long PTFE capillary (inner diameter 2 mm). A negative temperature gradient was applied along the last array by liquid nitrogen cooling; the others were kept at room temperature (21°C). Trace amounts of water caused formation of a thin ice layer at temperatures below −80°C (light blue). The temperature profile along the detection setup is shown by the blue dashed line. For clarity, only 16 detectors are shown per COMPACT detector array.

A pulsed (5 ms on, 15 ms off) ^48^Ca^+10^ ion beam from the GSI’s accelerator UNILAC irradiated a four-segment target wheel (Jäger et al., 2014) with ^244^PuO_2_ targets (average ^244^Pu thickness: 0.80 (1) mg/cm^2^) electro-deposited on 2.2 (1)-μm thick Ti foils (Runke et al., 2014). In total, 9.3 (9)⋅10^18 48^Ca ions passed through the targets. TASCA was filled with 0.8 mbar He gas and was operated in the High-Transmission Mode at a magnetic rigidity of Bρ = 2.27 Tm (Düllmann et al., 2010; Gates et al., 2011). The experimental setup downstream of TASCA is shown in Figure 1. It was similar to that described in (Yakushev et al., 2014). The EVaporation Residues (EVR) were spatially separated in TASCA from beam and unwanted nuclear reaction products by their differing magnetic rigidities (Bρ values) and were guided through TASCA to the TASCA focal plane, where they passed through the entrance window of the Recoil Transfer Chamber (RTC) (Even, et al., 2011). A 60 × 40-mm^2^ large window, separating the TASCA volume from the volume of the RTC, was made of a 3.3-µm thick Mylar foil and supported by a stainless-steel grid (80% transparency). The 20-mm deep RTC had the same cross section as the RTC entrance window, resulting in an inner volume of about 48 cm^3^. A purified He:Ar = 1:1 gas mixture at 1 bar filled the RTC and circulated in a loop at a gas flow rate of 2 L/min (33.3 cm^3^/s), forced by a membrane pump (MPC 101 Z, Welch™). The purities of the He and Ar gases were 99.9999% and 99.999%, respectively. The main reactive gas impurities, water and oxygen, were suppressed to a level of below a few ppm by the purification cartridges MC50-902FV and MC400-902FV (SAES™), which were installed in the loop. These purification cartridges had highly efficient internal particle filters, which removed aerosol particles with sizes down to 3 nm diameter. The detection setup comprised multiple arrays of the Cryo-Online Multidetector for Physics And Chemistry of Transactinides (COMPACT) (Yakushev et al., 2014). One COMPACT array consists of two rows of 32 Positive-Intrinsic-Negative (PIN) diodes each, mounted face to face, forming a narrow rectangular chromatography channel. They register α-particle energies and fission fragment energies of species undergoing nuclear decay inside the channel. A negative temperature gradient was applied along the last array by liquid nitrogen cooling; the others were kept at room temperature (21 °C). The setup used in the second run of the present work was similar to that described in the first Fl chemistry experiment at TASCA, consisting of two Au-coated COMPACT arrays (Yakushev et al., 2014). In the first run of the present work, a third COMPACT detector array, coated with SiO_2_, was added in front of the two Au-coated ones (Figure 1, dashed black rectangle).

The chemical behaviour of short-lived Hg, Pb, and Rn isotopes was studied in separate experiments at TASCA, under identical single-atom-at-a-time conditions (Lens et al., 2018). The separation of these chemical elements was achieved in COMPACT: i) the non-volatile metal Pb adsorbed on SiO_2_ in the first detector array; ii) the volatile metal Hg passed the first detector array and adsorbed in the second one on Au at room temperature; iii) partial deposition of the noble gas Rn occurred at the cold end of the last array (Figure 1). The transport efficiency from the RTC into COMPACT (62%) was measured for ^183^Hg (*T*_*1/2*_ = 9.4 s), by normalizing the rate of decays detected in COMPACT to that measured separately in a silicon detector mounted behind the RTC window (Lens et al., 2018). About 50% of the detected Hg atoms reached COMPACT within 0.4 s. The Hg deposition pattern on the Au film was measured in control experiments before and after each Fl experiment and remained unchanged.

## 3 Results and discussion

### 3.1 Observation of Fl isotopes in chemistry experiments at TASCA

The search for the signature of Fl atoms, i.e., nuclear decay chains registered inside the COMPACT arrays, was performed similarly to the previous work (Yakushev et al., 2014). The following search parameters were used to identify nuclear decays from Fl isotopes and their daughter nuclei: the energy window for the first α−decay was 9.4–10.3 MeV, the energy window for the subsequent α−decay was 8.8–9.4 MeV, the low energy limit for a spontaneous fission (SF) fragment was 20 MeV. The time windows for the search of correlated decay chains were 1 s for short decay chains (α_1_−SF) originating from ^288^Fl to 200 s for the long decay chains (α_1_−α_2_−SF) from ^289^Fl and ^285^Cn, respectively. For all SF decays with no preceding α-particles within those time and energy windows, a dedicated event-by-event search of all events was performed for a larger time window of 1000 s. In total, nine new decay chains were identified at low probabilities to be of random origin (see Table 1). Based on the known decay properties, six registered decay chains were assigned to two Fl isotopes (three to ^288^Fl and three to ^289^Fl), and three decay chains were assigned to the decay of ^285^Cn, the daughter nucleus of ^289^Fl. The probabilities for an observed decay chain to have random origin due to background was calculated using the given energy and time windows and considering the sum of the rates of six detectors: the detector pair (top and bottom), where the first alpha particle was registered, and the two neighbouring pairs. If the members of a decay chain were not found within three detector pairs (e.g. chain #2), the sum of rates for all detector pairs between the positions of the first and the last decay chain member was used for the calculation of the random probability. The temperatures at the detector positions, where the decays were registered, and the surface description are also given.

**TABLE 1:** Decay chains from ^288^Fl, ^289^Fl and ^285^Cn observed in chemistry experiments at TASCA.

Chain #	Decay assignment (energy in MeV^*^)	*Δt*_*1*_ [s]	*Δt*_*2*_ [s]	Random probability	Detector number^b^/Temperature	Surface
Run I	Beam integral 4.0(4)⋅10^18^					
1	α_1_(9.65)→SF(73 + 61)/^*288*^ *Fl →* ^*284*^ *Cn*	0.65	−	6.3⋅10^–6^	I-9/21°C	Au
2	α_1_(9.78)→α_2_ (9.11)→SF(37 + 78)/^*289*^ *Fl→* ^*285*^ *Cn→* ^*281*^ *Ds*	11.6	25.3	1.3⋅10^–6^	I-9 (α_1_)/21 °CII-20 (α_2_,SF)/−32°C	Au
						Au
Run II	Beam integral 3.1(3)⋅10^18^					
3	α_1_(**9.80**)→α_2_(**9.08**)→SF(**79 + 44**)/^*289*^ *Fl→* ^*285*^ *Cn→* ^*281*^ *Ds*	62.6	3.6	1.0⋅10^–6^	II-30/−134°C	Ice
Run III	Beam integral 6.2(6)⋅10^18^					
4	α_1_(**9.47**)→SF(**75 + 92**)/^*288*^ *Fl →* ^*284*^ *Cn*	0.22	−	1.6⋅10^–5^	I-2/21°C	Au
5	α_1_(10.02)→SF(**97**)/^*288*^ *Fl →* ^*284*^ *Cn*	0.45	−	3.1⋅10^–4^	II-29/−121°C	Ice
6	α_1_(**10.29**)→SF(**81 + 94**)/^*288*^ *Fl →* ^*284*^ *Cn*	0.18	−	8.3⋅10^–6^	I-7 (α_1_)/21°C	Au
					I-15(SF)/21°C	
7	α_1_(**9.70**)→α_2_(**9.31**)→SF(**86 + 22**)/^*289*^ *Fl→* ^*285*^ *Cn→* ^*281*^ *Ds*	78.4	39.7	9.1⋅10^–9^	II-30/−136 °C	Ice
8	α_1_(9.92)→α_2_(**9.28**)→SF(**68 + 84**)/^*289*^ *Fl→* ^*285*^ *Cn→* ^*281*^ *Ds*	80.0	40.3	1.9⋅10^–6^	I-13 (α_1_)/21°CI-31 (α_2_,SF)/21°C	Au
Incomplete chains^c^						
9	α_1_(9.15)→SF(67 + 50)/^*285*^ *Cn→* ^*281*^ *Ds*	25.0	−	6.2⋅10^–2^	I-8/21°C	Au
10	α_1_(9.15)→SF(**95 + 107**)/^*285*^ *Cn→* ^*281*^ *Ds*	1.0	−	2.5⋅10^–3^	I-8 / 21°C	Au
11	α_1_(8.89)→SF(93 + 124)/^*285*^ *Cn→* ^*281*^ *Ds*	0.8	−	4.2⋅10^–4^	II-7 / −7°C	Au

a Values for the α-decay and SF, events detected during beam-off periods are given in bold. The energies of SF, fragments are given as registered without any correction for the pulse height defect.

b The detector number reflects the number of the COMPACT, array and the number of the detector pair in the array from 1 to 32. The first Au-covered COMPACT, array is denoted as I; the second Au-covered COMPACT, array is denoted as II., Events #3 and #9 were observed in the experiment, where an additional SiO_2_-covered COMPACT, array was placed upstream of the two Au-covered COMPACT, arrays.

c The incomplete chains were observed during runs II and III.

**FIGURE 2:**
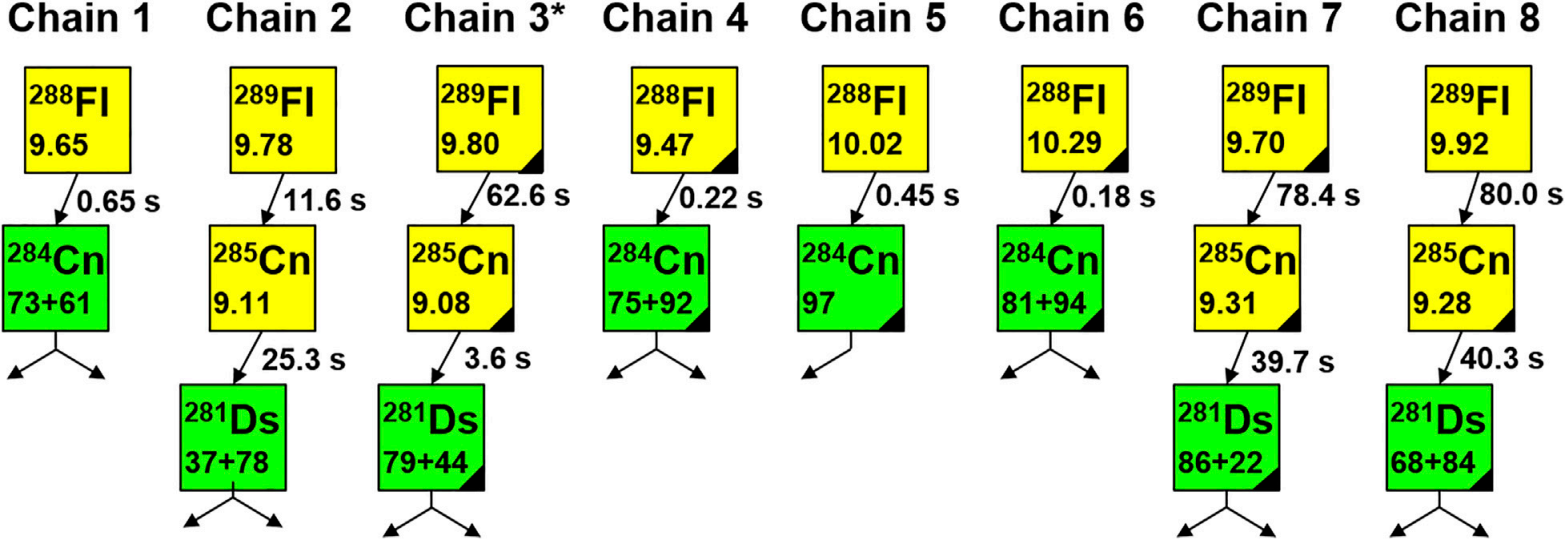
Decay chains originating from ^288,289^Fl registered in TASCA experiments. Chains 1 and 2 were reported in (Yakushev et al., 2014), chains 3 to 8 were observed in this work. Chain 3, marked with a star, was observed during the run with the SiO_2_-covered COMPACT array. Yellow squares correspond to α-decay, the green squares to SF decay. Measured energies of α particles and fission fragments are given in megaelectronvolts (MeV). SF energies are given without correction for pulse height defect. Black triangles denote decays registered during beam-off periods.

Six newly-observed complete decay chains are shown in Figure 2 (chains #3 to #8) together with the two decay chains observed in the first TASCA experiment (chains #1 and #2) (Yakushev et al., 2014).

The incomplete decay chains cannot be unambiguously assigned to a Fl or Cn deposition, recognising that Cn is also volatile and can be transported by the carrier gas after the decay of a Fl atom (Eichler et al., 2008; Wittwer et al., 2010; Yakushev et al., 2014). Also, seven SF events were found through the observation of both fission fragments in opposite detectors for each SF event, though, without α-decay precursors. No unambiguous assignment can be given for these SF events. Four SF events were registered in the first detector array, and they can be tentatively attributed to the short-lived ^284^Cn (T_1/2_ = $98_{-14}^{+20}$ ms), the daughter of ^288^Fl (Düllmann et al., 2010; Oganessian and Utyonkov, 2015; Såmark-Roth et al., 2021). Three SF events were registered in the last Au-covered COMPACT array. These events could originate from longer-lived species with a high volatility, such as Cn or Fl. However, no SF events from ^285^Cn and ^288,289^Fl have been observed to date. Thus, only the events where the position of the primary Fl α-decay was registered can contribute to a reliable interpretation of the chemical interaction of Fl with the detector surface.

The chemical information provided by the six Fl atoms observed in the present work was evaluated together with that of two Fl atoms reported in (Yakushev et al., 2014). The eight events form two zones (Figure 1). The first one, comprising five events, is located near the deposition zone of Hg. This suggests that those Fl species interact rather strongly with Au. On the other hand, three atoms passed the entire Au surface and were deposited at low temperature. These events form a second zone that overlaps with the Rn deposition zone, and, thus, is indicative of a weak interaction of the Fl−Au system. One of these was registered in the first run, where the SiO_2_-covered detector was placed in front of two Au-covered detectors, and thus, also passed a SiO_2_ surface kept at room temperature.

### 3.2 Monte Carlo simulations of the adsorption process

Single Fl atoms moving with the carrier gas along the detector arrays experience many collisions with the surface. At the surface, the measure of chemical reactivity can be described by the reaction rate constant k Eq. 1.

$$k\left(T\right)\;=\frac{k_{B}T}{h}\;\cdot \;exp\left(-\left(\frac{\Delta G}{RT}\right)\right)$$ (1)

where $k_{B}$ is the Boltzmann constant, h is the Planck constant, and R is the universal gas constant. The rate of the chemical reaction between a single Fl atom and a surface depends on the Gibbs free energy of activation (*ΔG*), temperature (*T*), and the number of collisions. In each surface collision, the adsorbed atom undergoes oscillation with the characteristic period $\tau_{0}$ . It is energetically trapped on the surface for a time *τ*_*a*_. This time depends on the activation energy needed for its desorption from the surface (*E*_*des*_) and on T , according to the Frenkel equation (Eq. 2) (Frenkel, 1948).

$$\tau_{a}\;=\;\tau_{0}\;\cdot \;exp\left(\frac{E_{des}}{RT}\right)$$ (2)

**FIGURE 3:**
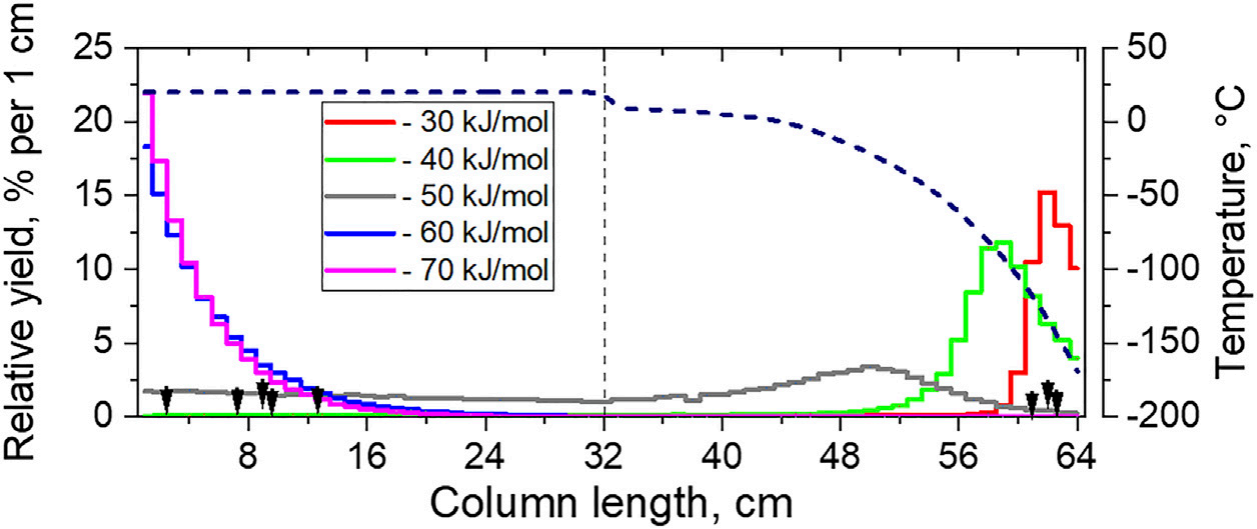
The simulated distributions of ^288,289^Fl in two Au-covered COMPACT detector arrays. The Fl deposition yields (in %) per individual pair of PIN diodes are shown as solid lines, simulated based on the original MC model for a given adsorption energy value. The positions of the observed Fl events are shown with black arrows. The temperature profile along the COMPACT detector arrays is shown as the dashed line.

Supposed that the energy needed for the desorption is equal to the negative adsorption enthalpy, $E_{des}=-\Delta H_{ads}^{Au}$ , MC simulations (MCS) of the gas-solid chromatography process based on the original model for mobile adsorption can provide spatial distributions of adsorbed species depending on their adsorption enthalpy values (Zvára, 1985). Such simulations were performed for the chromatography channel consisting of two Au-covered COMPACT arrays and for the experimental conditions described in the Experimental Section. Results from simulated distributions of Fl species obtained for five different adsorption enthalpy values are presented in Figure 3 together with the positions of the observed Fl events.

According to the simulations, Fl atoms adsorb almost quantitatively (>99%) in the first Au-covered COMPACT array if the adsorption enthalpy values are $-\Delta H_{ads}^{Au}\left(Fl\right)$ ≥ 60 kJ/mol. Five Fl events observed at room temperature in the first Au-covered COMPACT array suggest a rather strong binding of Fl atoms to gold. The probability to observe a Fl event at lower temperatures in this case is less than 1%. Thus, MC simulations performed for larger enthalpy values cannot describe the observation of three Fl events found on ice at a low temperature. The simulated distribution for a low adsorption enthalpy value of $-\Delta H_{ads}^{Au}\left(Fl\right)$ = 30 kJ/mol agrees well with the position of three Fl events found at the cold end in the second Au-covered COMPACT array and suggests a weak interaction. However, for this low adsorption enthalpy value, the expected number of Fl species deposited in the first COMPACT detector array is only about 2% and mostly due to decay in flight. Thus, the MC simulations based on the mobile adsorption mechanism performed for a single Fl species interacting with a homogeneous Au surface cannot describe the experimentally observed two-zone deposition of Fl in the COMPACT array.

The deposition patterns resulting from simulations of the mobile adsorption of a single Fl species on a homogeneous Au surface were evaluated for different values of the adsorption enthalpy (Figure 3). For high values of $-\Delta H_{\text{ads}}^{\text{Au}}\left(\text{Fl}\right)$ ≥ 60 kJ/mol, the simulated deposition patterns follow an exponential decay distribution due to the diffusion-controlled deposition. In particular, the deposition probability decreases along a detector array kept at a constant temperature due to the reduction of the total number of Fl atoms available for the process of mobile adsorption. For low values of $-\Delta {\text{H}}_{\text{ads}}^{\text{Au}}\left(\text{Fl}\right)$ ≤ 40 kJ/mol, a low-temperature peak is formed. The observed room-temperature deposition zone of Fl on the Au surface suggests conditions suitable for a stronger Fl interaction for the Fl−Au_bulk_ system and points at a rather high adsorption energy value valid for a chemisorption process. At lower temperatures, even weaker interactions, i.e., physisorption, can lead to the immobilization of Fl atoms due to retention times longer than the remaining nuclear lifetimes of the Fl atoms.

According to the simulations for the case of the mobile adsorption of a single Fl species on a homogeneous surface, the simultaneous observation of Fl in two different zones associated with a high and a low adsorption enthalpy is excluded. Thus, Monte Carlo simulations based on the mobile adsorption mechanism for a single chemical species interacting with a homogeneous surface (Zvára, 1985), as used in the previous works (Eichler et al., 2010; Yakushev et al., 2014), are unable to describe the observed Fl distribution. We note that this mechanism inherently assumes the kinetics of the surface interactions to be fast.

The experimental pattern can only result i) from the deposition of two different chemical species of Fl, or ii) from the presence of a surface inhomogeneity, providing sites of different reactivity towards Fl.

### 3.3 Potential chemical reactions of Fl in the gas phase within the RTC

The first case requires Fl to form a chemical compound. This appears most likely with a gas impurity (O_2_ or/and H_2_O) during the thermalization process inside the RTC volume, and it should also be valid for other elements, e.g., for Hg and Pb. The most probable Fl compound, which could be formed during the thermalization process is FlO (Liu et al., 2001). The stability of the metal-oxygen bond in monoxides decreases in the sequence PbO > HgO > FlO (Friswell and Jenkins, 1972; Liu et al., 2001; de Macedo et al., 2011). Both, Pb^0^ and PbO, are low-volatile species and should adsorb on SiO_2_ at room temperature (Lens et al., 2018; Yu et al., 2021). Thus, the deposition pattern of Pb is not elucidating whether PbO (or other compounds) formed. However, this is different for Hg. Preliminary results of periodic relativistic DFT calculations of the adsorption of the M and MO (M = Hg, Pb, Cn, Fl) species on the hydroxylated α−quartz surface suggest that HgO interacts much more strongly than Hg. The calculated adsorption enthalpy of HgO on the quartz surface $-\Delta H_{ads}^{quartz}\left(HgO\right)$ is larger than 200 kJ/mol (Pershina and Iliaš, 2022). Thus, if HgO is formed during the thermalization process inside the RTC, it should be deposited mainly in the first COMPACT array on the SiO_2_ surface due to chemisorption, similarly to other oxide surfaces, e.g. CaO and CeO_2_ (Ling et al., 2015; Zhao et al., 2018; Pershina and Iliaš, 2022). Studies of the adsorption properties of Hg, which can provide an indication on the formation of Hg compounds, were performed in separate experiments, where Hg species encountered first the SiO_2_ surface and then the Au surface (Lens et al., 2018). In these experiments, Hg did not adsorb on SiO_2_ in the first COMPACT array, but did so quantitatively on Au in the following one (Lens et al., 2018). This behaviour is indicative of the elemental form of Hg and is in line with that observed earlier (Eichler et al., 2010; Wittwer et al., 2010; Yakushev et al., 2014; Türler et al., 2015; Yakushev and Eichler, 2016) as well as in the present work. The formation of HgO during the thermalization process in the purified He-Ar gas mixture was neither observed in the present work nor in our preparatory studies (Lens et al., 2018). Thus, the formation of Fl compounds in the RTC, e.g., FlO, which are unanimously predicted to be less stable than those of Hg, is unlikely to occur. However, even though the formation of a Fl compound was not considered in the interpretation of any of the previous Fl chemistry experiments (Eichler et al., 2010; Wittwer et al., 2010; Yakushev et al., 2014; Yakushev and Eichler, 2016), this cannot be fully excluded.

### 3.4 Analysis of the Au layer structure on the detector surface

**FIGURE 4:**
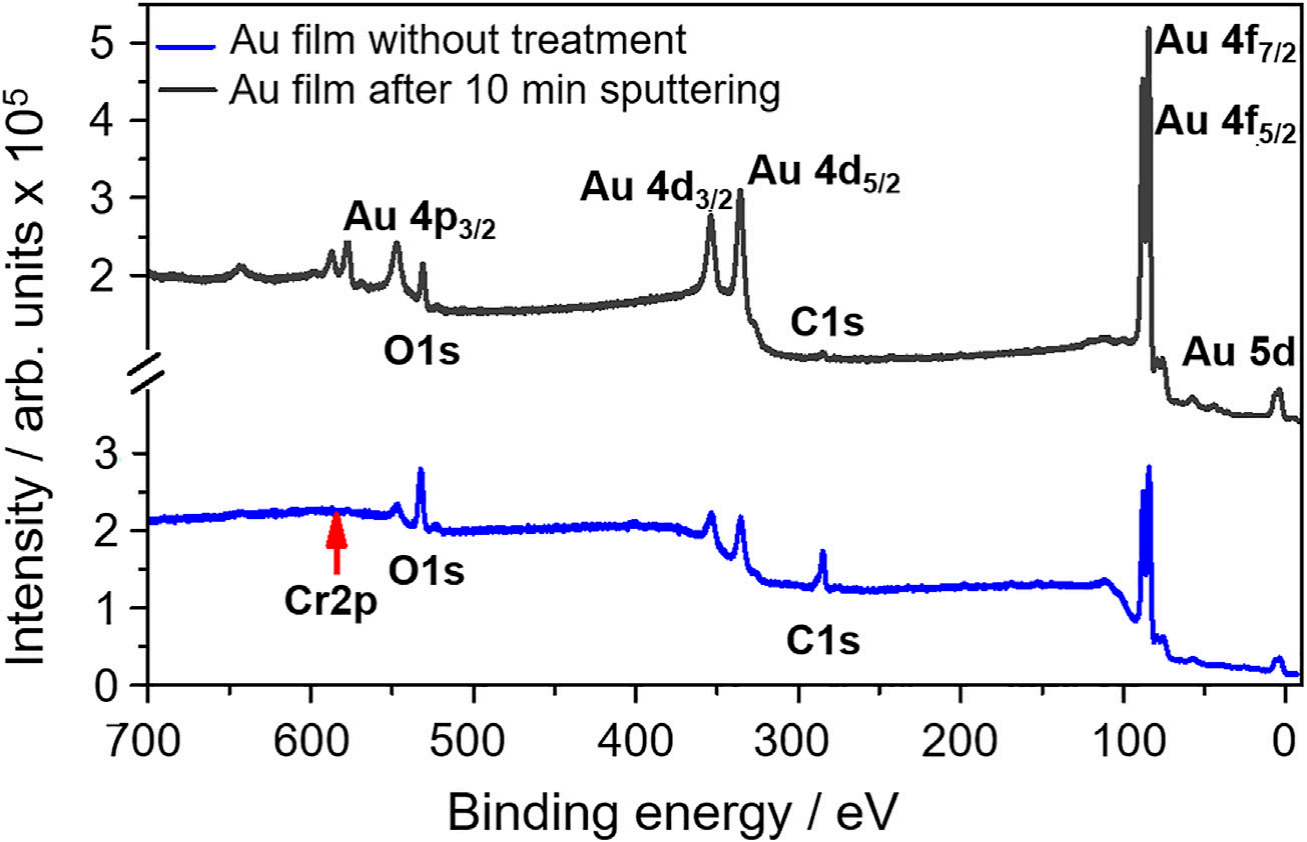
XPS spectrum of the Au-covered detector surface. The spectrum obtained without surface cleaning (blue) and the spectrum after 10-min cleaning by Ar-ion sputtering (black) are shown.

In the second scenario, the formation of two Fl deposition zones on Au is caused by the site and temperature-dependent chemical reactivity of Fl towards an inhomogeneous Au surface. The surface of Au-covered PIN diodes used in previous works (Eichler et al., 2008; Eichler et al., 2010; Wittwer et al., 2010; Yakushev et al., 2014; Lens et al., 2018), and in the present study consisted of a thin Au layer. The Au-coated surfaces of COMPACT detectors were produced by thermal evaporation of Au films onto Si(111) substrates. For the evaporated Au layer on top of the silicon detectors, an average thickness of about 50 nm was specified by the detector manufacturer (ITE, Warsaw). A detailed non-destructive study of the thin Au film on the detector surface was performed using several techniques: (i) scanning electron microscopy with energy-dispersive X-ray detection (SEM-EDX); (ii) X-ray photoelectron spectroscopy (XPS); (iii) atomic force microscopy (AFM); and, (iv) X-ray diffraction (XRD). The macro photographs, obtained with a scanning electron microscope at the target laboratory at GSI, Darmstadt, demonstrate the uniformness of the surface. The dominant peaks in the EDX spectrum measured with the same apparatus are from *M*_*α*_ and *M*_*β*_ lines of Au. The analysis for possible surface contaminations was performed using XPS. Several lines from *p*, *d,* and *f* electron shells of Au were clearly observed, but also some contaminations from light elements (C, O) (Figure 4). The carbon contamination disappears after 10-min surface cleaning by sputtering with an Ar ion beam, and the oxygen contamination is reduced. No other metals on the Au detector surface were observed.

**FIGURE 5:**
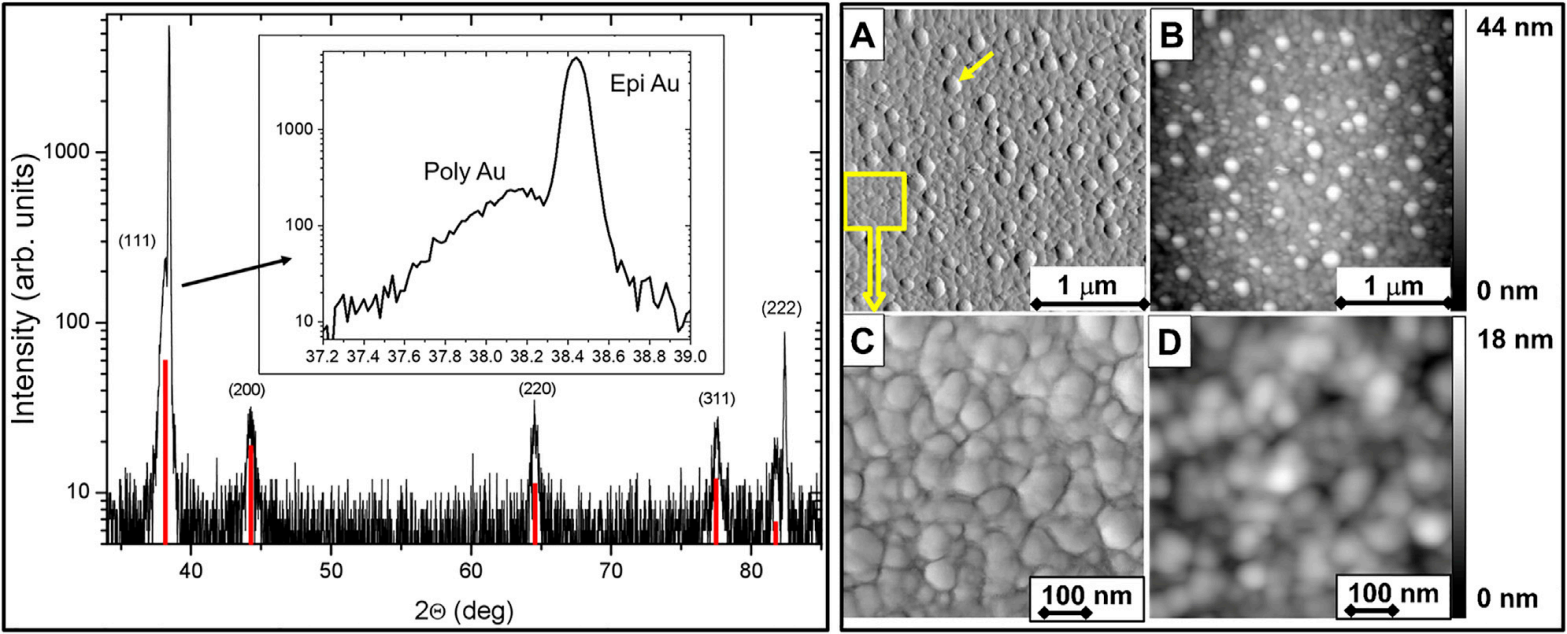
Left panel: XRD Θ/2Θ-scan of an Au film. The measured Θ/2Θ-scan of a thin Au film on top of a silicon detector (black line) and the simulated XRD intensity distribution for a polycrystalline Au film (red lines) are shown. One line corresponding to the Au (111) plane is expanded and shown in the inset. Right panel: Representative tapping mode AFM images of the gold surface on the detector show the phase contrast **(A,C)** and the topography **(B,D)** of the surface.

The XRD method was applied to clarify the crystalline structure of the Au film. The measured XRD Θ/2Θ-scan of the Au film is shown in Figure 5 (left panel), together with the simulated intensity distribution for a polycrystalline Au film (red lines). Several lines from different Au planes are clearly visible, in agreement with a simulated distribution for the polycrystalline Au film.

However, the Au (111) line is more intense than expected from a purely polycrystalline Au film, and consists of two components, see inset in Figure 5: a stronger one from epitaxial Au (111) and a weaker one from polycrystalline Au (111). The Au (222) line has two well separated peaks originating from epitaxial and polycrystalline Au, too. This analysis shows the presence of a mixture of polycrystalline and epitaxial Au (111) in the evaporated Au film. The topography of the Au-film surface was analysed by the AFM method. AFM measurements provided values for the size of Au crystallites and for the root-mean-square (RMS) roughness of the surface (Figure 5, right panel). The tapping mode is used for the acquisition of both, phase contrast (**A** and **C**) and topography (**B** and **D**) images. Au crystallites with grain boundaries can be clearly seen on phase contrast images, whereas the surface roughness can be determined from the topography images. Dispersed Au crystallites with extensions of about 100 nm (yellow arrow) protruding about 20 nm from the immediate fine-grained surrounding (yellow rectangle) are visible in the images A and B. The predominant part of the Au surface consists of Au grains with a size of 15–100 nm and with a roughness of about 4–5 nm. The enlarged view of the finer grains is shown on images C and D.

Based on the results of all applied analytical techniques, one can conclude that the Au film covering the silicon PIN diodes is inhomogeneous and consists mainly of polycrystalline Au grains with a mean grain size of about 70 nm; however, it also includes well-ordered epitaxial Au (111) crystallites. The mean grain size is close to the average Au layer thickness. Contaminants consisting of light elements (presumably adsorbed H_2_O and CO_2_) are present on the surface. No contamination from other metals was detected.

### 3.5 Adsorption of Fl on the inhomogeneous Au surface

**FIGURE 6:**
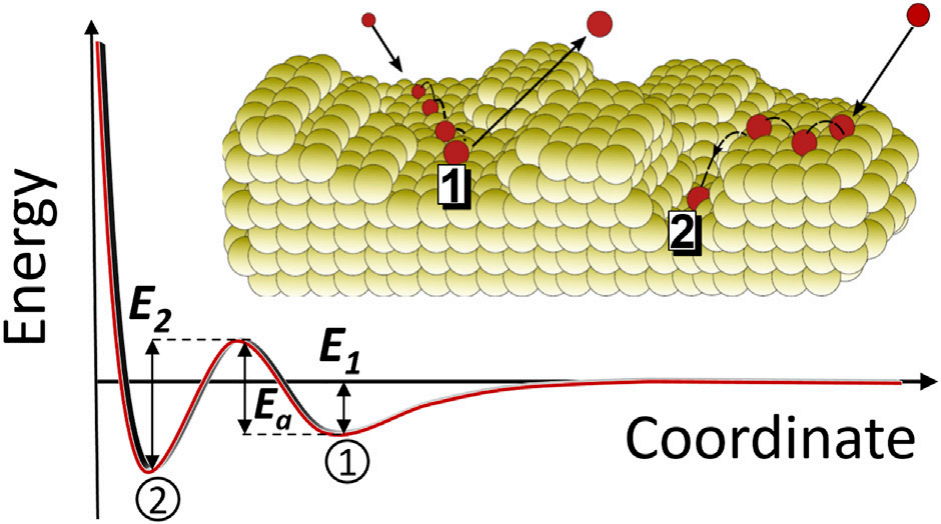
A simplified scheme of the adsorption mechanisms on an inhomogeneous surface, which includes the migration (diffusion) step from the physisorbed/precursor state (1) to a stronger-binding site (2), cf. Figure 4 in (Li et al., 2008).

The detector surface analysis revealed a morphologically inhomogeneous structure of the evaporated Au layer. This implies the surface to be also energetically inhomogeneous, offering a variety of sites with different potential energies. In addition, minor surface impurities were found. The following mechanism is proposed for the interaction with an inhomogeneous Au surface (cf. Figure 6). An impinging gas-phase atom (or, in a more general form, also molecule) becomes trapped in a shallow physisorption well of depth *E*_*1*_ via van der Waals interactions (Lennard-Jones, 1932). The atom/molecule stays in such a state for a time $\tau_{a}$ , during which it is mobile and can move on the surface. During this step, desorption from the physisorbed state back to the gas phase may occur (Figure 6, position 1). Alternatively, during diffusion the mobile atom/molecule may encounter a stronger-binding active site, e.g., a vacancy, a kink, a step, an impurity etc. (Figure 6, position 2). Radioactive species decay once their nuclear lifetime has elapsed; this can occur in either of the two trapped positions, or in the desorbed state (Zvara, 1985).

The physisorbed state may thus either correspond to the final state, or it may serve as a transitional state on the way to a more strongly bound state, reached, e.g., via diffusion. For this latter process to take place, an activation barrier of height *E*_*a*_ may have to be overcome (Ilbach, 2006). The surface residence time depends on the energy *E*_*1*_ needed for desorption and on the temperature (see Eq. 2 in chapter 3.2). If the atom/molecule overcomes the activation barrier and becomes more strongly bound, the residence time increases due to the higher energy that is required for desorption from this position. The time spent in the physisorbed/precursor state decreases with increasing temperature, however, the probability to overcome the activation barrier becomes larger in accordance with the Arrhenius expression (Hammer and Nørskov, 2000). Thus, this process of “activated” adsorption is favoured at higher temperatures.

According to this simple model, the fate of a Fl atom, physisorbed for a time $\tau_{a}$ on Au, will either be (i) desorption back into the gas phase, (ii) reaching a position where a more strongly bound state can be reached by jumping over an activation barrier of a height larger than the desorption energy (Figure 6), or (iii) decay in the physisorbed state. The “activated” stronger adsorption is favoured at higher temperatures due to a larger fraction of Fl atoms overcoming the activation barrier and decaying at an active site. Along the chromatography channel with its negative temperature gradient, this later fraction decreases with decreasing temperature, due to the increasing inability of atoms to overcome the activation barrier. In addition, the loss of the atoms that reached a strongly-binding position results in ever fewer atoms being available for deposition along the channel. Finally, adsorption of the fraction of Fl atoms, which never overcame the activation barrier at sufficiently high temperatures, occurs via the weak physisorption interaction at the low temperature end. The hypothetical Fl distribution formed by chemisorption at room temperature and by physisorption at a low temperature should have significantly enhanced intensities at the beginning of the column and at the cold end. In light of the very limited number of experimentally available atoms, Fl events were found only at the two deposition zones, where enhanced intensities are expected. Thus, our observation is consistent with this model.

## 4 Conclusion

In summary, the chemical character of Fl has been further elucidated based on the observation of its interaction with SiO_2_ and Au surfaces. The Fl events were distributed over two deposition zones: five atoms form the first one at room temperature; three atoms passed the entire Au surface and were deposited at low temperature on ice at the cold end. One of these latter three atoms also passed a SiO_2_ surface kept at room temperature before encountering the Au surface. We assign the behaviour of these three atoms to the transport of Fl in the elemental form. This observation is similar to that for two out of three atoms reported in ref. (Eichler et al., 2010). The remaining five atoms did not reach the cold end of the chromatography column but were already adsorbed on Au surfaces kept at room temperature, similarly to the third atom reported by (Eichler et al., 2014) and to two atoms reported by (Yakushev et al., 2014 ).

Evidently, two types of interaction of a Fl species with the detector surfaces were observed in the present work − chemisorption on Au at room temperature, and physisorption on ice − suggesting that Fl exhibits lower reactivity towards Au than the volatile metal, Hg, but is capable of forming stronger bonds with Au than the noble gas Rn is. Two scenarios are proposed to explain the experimental observation. In the first scenario, the formation of a Fl compound (e.g. FlO), which can be more reactive towards Au than elemental Fl, is considered but is viewed as an unlikely option under the given experimental conditions. In the second scenario, a stronger binding of Fl atoms on energetically favoured sites of the inhomogeneous Au surface is proposed, which includes the transition of a Fl atom over an activation barrier. Further support for this scenario would benefit from increased statistics, as the current level did not allow to also observe evidence for the small fraction of all events that is expected to be present between the two deposition zones according to this model. Both described scenarios, though, imply that Fl forms chemical bonds, and both are able to describe the complete experimental data set available on chemical properties of Fl: our new data presented here as well as those published in (Eichler et al., 2010; Yakushev et al., 2014). Based solely on the currently available experimental data, an unambiguous statement in favour of one scenario over the other cannot be concluded. Further investigations are desirable to shed more light on Fl reactivity in the gas phase and its interactions with different surfaces.

## Data Availability

The raw data supporting the conclusion of this article will be made available by the authors, without undue reservation.
